# Role of TREM-1 in endothelial dysfunction during experimental sepsis

**DOI:** 10.1186/cc11736

**Published:** 2012-11-14

**Authors:** A Boufenzer, N Sennoun, Y Bouazza, M Derive, PE Bollaert, S Gibot

**Affiliations:** 1 Groupe CHOC - Inserm U961, Nancy University, Nancy, France; 2 CHU Nancy, France

## Background

Triggering receptor expressed on myeloid cells-1 (TREM-1) is a receptor of the immunoglobulin superfamily expressed on the surface of neutrophils and monocytes/macrophages. It plays an important role during sepsis by amplifying the inflammatory response. Modulation of TREM-1 through the administration of a short synthetic peptide (LR12) increases survival during experimental sepsis. This study aimed to explore the mechanisms by which LR12 prevented sepsis-induced cardiovascular dysfunction.

## Methods

We studied the effect of TREM-1 modulation by a synthetic peptide LR12 (3 mg/kg) on MAP and blood lactate during experimental sepsis (CLP). Aortic and mesenteric arterial vessels of these animals were collected to study the vasoreactivity to phenylephrine (Phe) and acetylcholine (Ach) *ex vivo *(Myograph). Alternatively, vasoreactivity was studied in the vessels of healthy animals (with and without endothelial lawyer) stimulated with LPS or with a specific agonist of TREM-1 (αTREM-1, 10 μg/ml), with or without LR12 (20 mg/ml). The effect of LR12 on arterial vessels was also studied through western blotting (eNOS, iNOS, Akt, COX-1, COX-2) and qRT-PCR. Mouse lung microvascular endothelial cells (LUMECs; CD146^+^) were analyzed by flow cytometry, qRT-PCR, and ELISA to decipher the effect of LR12 on LPS-induced endothelial activation.

## Results

CLP induced MAP decrease and lactate elevation were prevented by the administration of LR12. Arterial vessels from septic animals treated with LR12 showed better response to Phe and Ach compared with controls. The reactivity of aortic and mesenteric vessels (contraction and relaxation) stimulated *in vitro *with LPS or by αTREM-1 was altered: this phenomenon was reversed by LR12. LR12 restored the phosphorylation of Akt and eNOS while it reduced the activation of inducible pathways (iNOS, COX-2). FACS analysis showed that TREM-1 is constitutively expressed by LUMECs (CD146^+^/VEGFR2^+^). *In vivo*, the expression of *Trem-1 *was increased in septic animals and was inducible *in vitro *upon stimulation with LPS. *Trem-1*, *Tnf-α *and *Il-6 *expression was upregulated by LPS; once again LR12 attenuated this upregulation. Finally, the production of several cytokines by LPS-stimulated LUMECs was decreased by LR12.

## Conclusion

Here we show that TREM-1 is expressed on endothelial cells from the aorta, mesenteric artery, and microvascular endothelial cells, and that TREM-1 might be directly involved in endothelial dysfunction during experimental sepsis.

